# Augmented visual feedback counteracts the effects of surface muscular functional electrical stimulation on physiological tremor

**DOI:** 10.1186/1743-0003-10-100

**Published:** 2013-09-24

**Authors:** Giuliana Grimaldi, Alfredo Fernandez, Mario Manto

**Affiliations:** 1 Unité d’Etude du Mouvement (UEM), ULB-Erasme, 808 Route de Lennik, 1070, Bruxelles, Belgium

**Keywords:** Functional electrical stimulation, Augmented visual feedback, Physiological tremor, Power spectra

## Abstract

**Background:**

Recent studies suggest that surface muscular functional electrical stimulation (FES) might suppress neurological upper limb tremor. We assessed its effects on upper limb physiological tremor, which is mainly driven by mechanical-reflex oscillations. We investigated the interaction between FES and augmented visual feedback, since (a) most daily activities are performed using visual cues, and (b) augmented visual feedback exacerbates upper limb tremor.

**Methods:**

10 healthy subjects (23.4 ± 7.7 years) performed 2 postural tasks with combinations of FES (4 sites; frequency of stimulation: 30 Hz; pulse width: 300 microsec; range of current delivered 10–34 mAmp) and augmented visual feedback.

**Results:**

Spectral analysis of tremor showed a decrease of power spectral density to 62.18% (p = 0.01), of the integral in the 8-12 Hz frequency band to 57.67% (p = 0.003), and of tremor root mean square (RMS) to 57.16% (p = 0.002) during FES, without any changes in tremor frequency. Augmented visual feedback blocked the beneficial effect of FES, as confirmed by power spectral analysis (p = 0.01). We found a statistically significant interaction between augmented visual feedback and electrical stimulation (p = 0.039).

**Conclusions:**

Augmented visual feedback antagonizes the effects of FES on physiological tremor. The absence of changes of peak frequency argues against an effect of FES on mechanical properties of the upper limb.

## Introduction

Tremor is a non linear and non stationary phenomenon, also defined as a rapid back-and-forth movement of a body part [1,2]. Physiological tremor (PT) is an involuntary rhythmical movement of limb segments typically in the frequency range of 8-12 Hz, with a small amplitude barely visible to the naked eye [3].

PT has two components: mechanical-reflex oscillations and central-neurogenic oscillations [4,5]. Mechanical-reflex oscillations are invariably present and represent the most noticeable contribution to the genesis of the rhythmic activities of the limbs [6]. Oscillations are characterized by a frequency governed by the inertial and elastic properties of the tremorous body segment [4]. Mechanical-reflex tremor occurs in response to irregularities in muscle contraction, vibrations produced by cardiac systole, and external perturbations (e.g., someone bumping the limb) [7]. The frequency (ω) of these passive mechanical oscillations depends directly upon the stiffness (K) of the joint and inversely upon the inertia (I) according to the following equation [6]:

$$\omega \;=\;\surd \;\left(K/I\right)$$

Consequently, tremor frequency increases from proximal to distal segments: tremor of the elbow has a frequency of 3-5 Hz, wrist tremor 7-10 Hz and metacarpophalangeal joint tremor 12-30 Hz [5]. Another direct consequence of this law is a decrease of frequency when an inertial load is added to the limb. Regarding central neurogenic oscillations, they are associated with a modulation of motor unit activity, and occur at frequencies of 8-12 Hz and 15-30 Hz. They are not dependent of limb mechanics (inertia and stiffness) or reflex loop time, and consequently are not influenced by inertial loading.

The effects of usual vision or augmented visual feedback (the subject has to maintain hand in a restricted position using reinforced visual feedback) on tremor have been examined earlier [8,9]. When usual visual feedback is removed, no change occurs in tremor magnitude as compared to the condition of normal vision [9]. For PT associated with isometric contractions, the magnitude is not influenced by usual vision [8]. Healthy subjects show a reduction in the magnitude of PT when visual feedback is delayed [10]. By contrast, augmented visual feedback increases the magnitude of tremor. This increase is the consequence of: (1) the effects of the interactions between vision and motor activity upon motor cortex excitability, with an enhanced excitability of the motor cortex during visuomotor tasks [11], (2) the increased muscle activity due to subject’s attempts to reduce tremor [12].

It has been proposed that surface muscular functional electrical stimulation (FES) represents an alternative approach to drugs or surgery to reduce neurological tremor [13,14]. However, the mechanisms of action of FES in neurological tremor are unclear. They remain difficult to investigate given the complexity of the mechanisms of tremor when the nervous system is affected, the heterogeneity of the various forms of tremor encountered during clinical practice, the interactions with drugs and the fluctuations of tremor as the neurological disorder progresses with time. One hypothesis is that FES increases the stiffness of the joints in neurological patients, and thus reduces tremor magnitude by changing the mechanical properties of the limb [15]. To our knowledge, the effects of FES on PT and the interactions with augmented visual feedback have not been investigated so far. Given the importance of passive mechanical oscillations in the genesis of PT, this study could thus provide novel insights into the mechanisms of action of FES on tremor.

The aims of the present study were: (1) to test the hypothesis that FES decreases the amplitude of PT, (2) to assess the effects of FES on tremor frequency (a change of peak frequency would argue for an effect of FES on mechanical properties of the limb), and (3) to assess the effects of FES on PT in a condition of augmented visual feedback. Furthermore, in order to obtain a direct measurement of the impact of FES on the mechanical properties of the limb, we also investigated the effects of FES on wrist’s stiffness using a robotic technique. Moreover, to obtain a confirmation that augmented visual feedback increases tremor magnitude by a direct modulation of the central nervous system pathways, we investigated the effect of Anodal continuous Direct Current Stimulation (Anodal cDCS) applied over the cerebellum on PT in the condition of augmented visual feedback. Indeed, (a) Anodal cDCS enhances the cerebellar cortical activity and thus increases the cerebellar-brain inhibition, resulting in a decrease of motor cortex excitability, and (b) cerebellum plays a determinant role in the modulation of tremor [16,17].

### Subjects and methods

#### Subjects description

We enrolled 10 healthy subjects (Males/Females = 8/2; mean age = 23.4 ± 7.7 years) following approval by the Ethical Committee of ULB. Subjects signed a written consent following full explanation of the experimental procedures. We used the following inclusion criteria: healthy males and females, no medication and no regular alcohol intake. The exclusion criteria were: history of brain trauma, metabolic disorders (diabetes, hyperthyroidism), history of weakness of the upper limbs, sensory disturbances, skin diseases affecting the arms, prolonged deprivation of sleep or food the day before the assessment, caffeine intake, pregnancy. To assess the dominance of the hand, we used the Edinburgh Handedness Inventory [18]. The scores ranged from -37.5 to -100 (mean = -78.48; median = -80), except for one subject who was left-handed (score = +68).

#### Mechanical counter test (MCT)

In order to confirm that the subjects included did not exhibit any impairment in hand dexterity and that the sample of subjects was homogeneous in terms of motor performances in upper limbs, we tested their motor capacity using a validated mechanical counter test (MCT) [19]. The subjects were comfortably seated in a quiet room. Two procedures were applied in each upper limb: a task of clicking repeatedly with the thumb on a single mechanical counter (MCT-U; upper limb maintained at rest during the task), and a task of clicking alternatively with the index finger on 2 mechanical counters in the horizontal plane (MCT-A) [19]. For each side, 3 practice trials of 10 seconds were applied before assessment, followed by 3 assessments at 10 sec, 20 sec and 30 sec, respectively (9 measurements for each upper limb in each of the two procedures). A rest of 15 seconds was applied before each measurement to avoid muscle fatigue. A highly linear increment in both MCT-U and MCT-A performances is expected for control subjects without sensorimotor disturbances.

#### FES

The effects of FES on tremor were studied only in the dominant upper limb (on the right side: n = 9 subjects; on the left side: n = 1 subject). Subjects were equipped with:

-4 triaxial accelerometers (Biopac, USA) affixed with adhesive tape respectively at the level of dorsal part of the index finger (3rd phalanx, accelerometer 1), dorsal part of the hand (at the level of the second metacarpal bone, accelerometer 2), elbow (about 2 cm below the olecraneum, accelerometer 3), shoulder (below the acromion, accelerometer 4). The accelerometers were calibrated and the calibration was checked throughout the experiments (1.55 mV = 100 mm/sec^2^).

-4 sets of surface electrodes: disposable adhesive electrodes (Ambu Neuroline 700, Denmark) were fixed on the following muscles in a belly-belly method (distance between electrodes: 3 cm; impedance of electrodes < 1KOhms): (1) flexor radialis carpi, (2) extensor radialis carpi, (3) biceps muscle, (4) triceps muscle. The selection of these sites is based on previous findings in neurological patients and on biomechanical models of simulation of the effects of FES on upper limb tremor [15,20-22]. For electrical stimulation of the skin, we applied trains (ramp mode: rising phase of 1 sec, plateau of 20 sec, decreasing phase of 1 sec; pulse width of 300 microsec) at a frequency of stimulation of 30 Hz (stimulator EM4PRO; Schwa Medica, France). The selection of these parameters of stimulation was based on previous studies [21,22].

**Figure 1:**
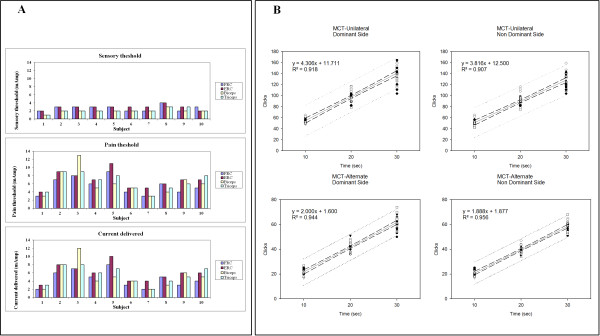
**FES parameters and hand dexterity. A:** current delivered on the skin in the 10 subjects for each of the 4 muscles. Top panel: ranges of sensory threshold. Middle panel: pain thresholds. Bottom panel: intensities of current delivered during FES. Values are expressed in mAmp. Abbreviations: FRC: flexor radialis carpi; ERC: extensor radialis carpi. **B:** hand dexterity evaluated with the Mechanical counter test (MCT), dominant side on left panels and non dominant side on right panels. Top panels: results of MCT- Unilateral (MCT-U). Bottom panels: results of MCT-Alternate (MCT-A). The increment in number of clicks was linear as a function of recording time, as confirmed by the regression analysis. Dashed lines: 99% confidence interval; dotted lines: 99% prediction intervals.

We first determined the sensory thresholds to exclude any sensory deficit in our subjects. For each of the 4 sites of stimulation, we increased the intensity by increments of 1 mAmp until subjective perception of the trains of stimuli on the skin (Figure 1A). Ranges of sensory threshold were 6-14mAmp (total amount of current delivered on the upper limb). Pain thresholds (defined as the first painful sensation during the incremental assessment) ranged from 14 to 38 mAmp (total amount of current delivered on the upper limb). We thus selected a range of intensities of current delivered just below the triggering of the painful sensation (10-34 mAmp). The selection of intensity delivered was thus based on subjects’ tolerance [15,21,22].

#### Augmented visual feed-back

In order to assess the effects of augmented visual feedback, a home-built light laser pointer was affixed to the palmar side of the index finger of the dominant arm. The laser was fixed below a rigid light plate affixed on the palmar side of the hand to avoid flexion of fingers or movement at the level of the metacarpophalangeal joints [23]. When the laser was switched on, the subject was asked to maintain the laser light within a black circle of 3 mm of diameter (drawn on a white background), located horizontally in front of the hand at a distance of 25 cm. This task requires continuous attention by subjects to maintain the laser light in the middle of the target circle. One investigator was seated near the subject to ensure that the task was correctly performed. Recording was excluded if the laser light left the black circle without an immediate return as assessed by visual inspection (3 trials amongst a total amount of 400 recordings –see below- had to be excluded and were collected once again). The selection of size of the diameter of the target and the distance hand/laser target is based on our previous experiments with fixed targets during visually-guided movements.

#### Tasks H and A

PT was investigated during successive postural tasks executed (a) without or with augmented visual feedback, and (b) without or with application of FES. Subjects executed the following 4 tasks:

(1) H: upper limb maintained outstretched and motionless, horizontally at the level of the shoulder, parallel to the floor. The hand had to be kept in pronation along the axis of the forearm;

(2) A: forearm fully supported and affixed on an armchair with a belt and tape, so that motion of the hand was restricted to the wrist. Subjects had to maintain the hand extended horizontally parallel to the floor (in pronation) and motionless.

In each of these 2 tasks, we thus studied 4 successive conditions: NSNL (stimulation off and laser off), NSL (stimulation off and laser on), STINL (stimulation on and laser off), STIL (stimulation on and laser on). We did not apply a random order for these 4 conditions on the basis of our previous experience on recordings of PT and FES with neurological patients, showing an absence of learning effect. In each of these 4 conditions, 5 recordings of 15 sec were performed (5 × 4 = 20 recordings per subject and per task; total of 200 × 2 = 400 recordings for the 10 subjects).

#### Signal processing

The sampling rate was 512 Hz per axis and per accelerometer. For signal processing of accelerometry signals, we computed the Root Mean Square (RMS) and performed the spectral analysis using Fast Fourier Transform (FFT) as recommended, using Matlab (MathWorks, USA) [2,24,25]. The 15 sec time-series were segmented in 5 segments. Auto-spectra of 5 sequential 3 sec data epochs were averaged to produce smoothed autospectra, with mean removal and a windowing (Hamming) for each data segment [24]. The following parameters were extracted and means (for the conditions NSNL, NSL, STINL, STIL) were computed: maximal PSD (maxPSD), peak frequency of power spectra (PFr), crest factor in the 4-20 Hz frequency band (CF = the ratio of maximal PSD divided by the integral of the 4-20 Hz frequency sub-band), Integrals of frequency sub-bands (4-8 Hz, 8-12 Hz, 12-16 Hz, 4-20 Hz, 20-30 Hz, 30-40 Hz), frequency dispersion (frequency width of the interval around the center frequency that contains 66% of the total power spectrum) [2]. Data from each axis of each accelerometer (cartesian coordinates) and the composite data (square root of the sum of the accelerations squared for all three axis) were processed as reported earlier [26]. For task H, we analyzed data recorded from the 4 accelerometers. For task A, we analyzed data from accelerometers located at index finger and wrist only, since the forearm/upper arm movements were prevented. In the results section, the data related to accelerometer 1 (the most relevant for PT assessment) [5,6] will be presented in details. Results related to the other accelerometers are given in the Additional file 1.

#### Investigation on the effect of FES on upper limb stiffness

We tested the effects of FES on the mechanical properties of the limb by measuring directly wrist stiffness before and during FES. We conducted a detailed single-subject experiment employing a real time mechatronic myohaptic unit device [27]. The subject (right-handed) was confortably seated with the dominant forearm fixed on the manipulandum. We measured wrist’s stiffness (expressed in Nm/rad) during a horizontal wrist extension imposed by the myohaptic unit at a slow speed (0.02265 rad/sec). This method is based on previous studies demonstrating that stiffness and gravity are the principal biomechanical factors of the neuromuscular system for slow motion [28]. We recorded 20 trials at baseline and 20 trials during FES. Stimulation was delivered at flexor radialis carpi, extensor radialis carpi, biceps muscle, and triceps muscle. Current intensity was 7 mAmp, 8 mAmp, 6 mAmp, 5 mAmp, respectively (sensory threshold was 3 mAmp, 4 mAmp, 5 mAmp, 3 mAmp, respectively. Pain threshold was 8 mAmp, 9 mAmp, 7 mAmp, 6 mAmp, respectively. For the stimulation pattern and details, see the section on FES above).

#### Investigation on the central effects of augmented visual feed-back effect: cerebellar contribution

We studied the effects of Anodal cDCS of the cerebellum on accelerometry in one right-handed subject confortably seated with the forearm fixed on the armchair and who executed a laser pointing task (augmented visual feedback) in 4 conditions: eyes open no stimulation (condition 1), eyes closed no stimulation (condition 2), eyes open post-cDCS (condition 3), eyes closed post-cDCS (condition 4). In the eyes open conditions (1 and 3), the subject used the laser to point towards the target (see above the augmented visual feed-back section). The rationale to study the effects of Anodal cDCS of the cerebellum is that (1) the cerebellum plays a critical role in the central modulation of tremor [29], and (2) Anodal cDCS of the cerebellum enhances the activity of the cerebellar cortex and thus decreases the excitability of the motor cortex [30].

Anodal cDCS was delivered on the right cerebellar cortex using a sponge electrodes (size 50×40 mm, soaked with a solution of NaCl 0.9%) [17]. The anode was located in the area between inion and mastoid, the cathode was located over the contralateral supra-orbital area. The period of stimulation lasted 20 min. Current delivered was 1 mAmp (portable simulator with a 9 V battery; CES, Canada). Current was increased gradually from 0 to 1 mAmp over 30 s, as confirmed by the analysis of the current using a Fluke PM3384A Combiscope. In each of the 4 conditions, 5 recordings of 15 sec were performed and PT was recorded with a tri-axial accelerometer located at the index finger. The sampling rate was 512 Hz. We computed the RMS and performed the spectral analysis. The following spectral parameters were extracted and means were computed: maxPSD, PFr, CF, Integrals of frequency sub-bands (4-8 Hz, 8-12 Hz, 12-16 Hz, 4-20 Hz, 20-30 Hz, 30-40 Hz), center frequency (for details, see the section signal processing above).

#### Statistical analysis

We used Sigma Stat (Jandel Scientific, Germany) to evaluate the statistical significance. A linear regression was applied to assess the data of the MCT tests. Linear regression was evaluated for each subject in each of the 4 sets of measurements (MCT-U and MCT-A on both sides), as well as for the whole group of subjects. For tremor data, normality of data was assessed with the Kolmogorov-Smirnov test. We looked for an effect of FES and augmented visual feedback on PFr, maxPSD, frequency bands of power spectra (Integrals), and on RMS. We applied the repeated measures analysis of variance (ANOVA on parametric data) or the ANOVA on ranks, according to the results of the normality assessment, followed by post-hoc test (Tukey test). We assessed the stimulation effect, the vision effect, and the interaction stimulation by vision effect. In order to better understand the effectiveness of FES in terms of tremor reduction, we also studied the relationship between the sum of intensities of current delivered at the 4 skin sites and the reduction on maxPSD induced by stimulation in the condition laser off (STINL). Data of the 10 subjects were fitted with a polynomial regression. To compare the stiffness of the wrist measured with the myohaptic unit before and during FES, we used a Student t test. To assess the effects of Anodal cDCS of the cerebellum on maxPSD of tremor, we expressed the data as means +/- SD and we computed a z score as compared to baseline. Statistical significance was set at 0.05.

## Results

### Mechanical counter tests

Data and results of linear regression analysis are given in Table 1 and shown in Figure 1B. In the MCT-U test for the dominant side, none of the subjects had values below 25 for the 10 seconds’ measurement. As expected for control subjects without sensorimotor impairments, the increment in the number of clicks was linear as a function of recording time on both sides. In the MCT-A test for the dominant hand, all the subjects reached a number of clicks >15 for the 10 seconds period. The increment in number of clicks was also linear as a function of recording time on both sides as demonstrated by the regression analysis.

**Table 1:** **Mechanical Counter Test data and regression analysis results (R**^**2**^**)**

**MCT-U dominant hand**										
***Subject***	***1***	***2***	***3***	***4***	***5***	***6***	***7***	***8***	***9***	***10***
**T10**	53	57	49	57	59	49	57	65	55	53
	57	55	57	52	56	49	53	62	52	52
	54	54	59	50	53	49	51	54	53	50
**T20**	103	102	117	102	104	89	83	116	97	97
	99	97	112	105	101	79	90	112	97	90
	100	101	108	101	102	90	92	108	98	94
**T30**	145	151	165	148	149	129	121	159	143	135
	148	150	159	139	141	115	111	147	144	131
	144	154	163	134	150	120	104	141	142	125
**R**^**2**^	0.99	0.99	0.99	0.98	0.99	0.97	0.96	0.96	0.99	0.99
**MCT-U non dominant hand**										
***Subject***	***1***	***2***	***3***	***4***	***5***	***6***	***7***	***8***	***9***	***10***
**T10**	44	48	53	49	50	43	43	47	45	49
	51	49	49	43	49	45	45	49	40	47
	45	52	51	44	45	42	45	46	43	46
**T20**	81	99	93	86	89	81	88	86	74	87
	99	97	90	86	79	78	87	83	73	85
	92	98	90	85	79	75	85	77	71	87
**T30**	134	139	138	120	115	110	135	131	105	124
	119	132	133	109	117	112	127	113	116	118
	126	135	132	118	124	110	128	130	118	112
**R**^**2**^	0.96	0.99	0.99	0.98	0.98	0.99	0.99	0.96	0.97	0.98
**MCT-A dominant hand**										
***Subject***	***1***	***2***	***3***	***4***	***5***	***6***	***7***	***8***	***9***	***10***
**T10**	21	21	22	18	22	24	24	22	20	17
	20	26	22	21	21	22	24	22	21	18
	23	21	22	21	21	27	25	21	20	21
**T20**	43	41	42	36	41	45	44	47	42	36
	36	40	45	38	42	46	41	44	38	38
	36	45	51	38	42	48	44	41	40	38
**T30**	50	64	68	58	63	70	65	65	57	59
	53	62	65	55	63	70	64	64	61	59
	57	64	66	54	64	74	60	61	56	61
**R**^**2**^	0.96	0.98	0.98	0.99	0.99	0.99	0.99	0.98	0.98	0.99
**MCT-A non dominant hand**										
***Subject***	***1***	***2***	***3***	***4***	***5***	***6***	***7***	***8***	***9***	***10***
**T10**	19	22	18	19	20	22	20	24	15	20
	18	21	21	18	22	25	20	22	17	22
	21	19	23	18	21	22	20	19	18	23
**T20**	37	39	38	37	41	38	40	38	33	38
	35	43	40	37	40	38	41	37	37	40
	36	44	36	34	40	41	40	39	36	39
**T30**	54	57	58	52	59	62	61	56	54	55
	51	59	54	53	62	54	61	57	57	53
	53	59	55	53	62	68	57	63	51	57
**R**^**2**^	0.99	0.98	0.98	0.98	0.99	0.94	0.99	0.97	0.98	0.99

### Effects of FES on physiological tremor and interaction with augmented visual feed-back

**Figure 2:**
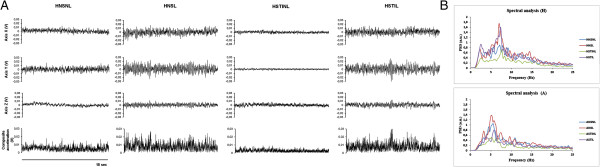
**Tremor recordings and inherent power spectra. A:** Example of recordings in subject 2, obtained with triaxial accelerometry. Signals (duration of epochs: 15 sec) recorded at the level of the index finger during task H in the 4 conditions (NSNL: no stimulation, laser off; NSL: no stimulation, laser on; STINL: stimulation off, laser off; STIL: stimulation on, laser on). Axis x, y and z (cartesian coordinates) and composite data are shown for each condition. A strong reduction of tremor oscillations is observed in HSTINL as compared to HNSNL and HNSL. The reduction is antagonized by augmented visual input (HSTIL). **B:** power spectra (related to tridimensional composite data; expressed in arbitrary units: a.u.) obtained by FFT analysis of tremor recorded at the finger during tasks H (Top panel) and A (Bottom panel) in the 4 conditions. Note the reduction of power spectra in the condition STINL (green lines).

Figure 2A illustrates a typical example of the effects of FES on tremor recorded at the index finger in one subject for position H. The 4 conditions are shown (HNSNL, HNSL, HSTINL, HSTIL). In absence of electrical stimulation, augmented visual feedback tended to enhance slightly amplitude of tremor oscillations (see HNSL as compared to HNSNL). A clear reduction of the magnitude of oscillations was observed during stimulation in absence of augmented visual feedback (HSTINL). Interestingly, this subject spontaneously reported a beneficial effect of FES in terms of stabilization of the hand during this latter condition (3 other subjects also perceived a benefit). Interestingly, this effect was antagonized by augmented visual feedback (HSTIL). Again, this subject perceived that augmented visual feedback induced a disappearance of the beneficial effects of electrical stimulation. Similar observations were made for position A. The observations above were confirmed by the detailed spectral analysis. Figure 2B shows the results of the FFT analysis (composite acceleration – accelerometer 1) for both tasks H and A in this subject. No obvious change in terms of PFr could be detected.

**Figure 3:**
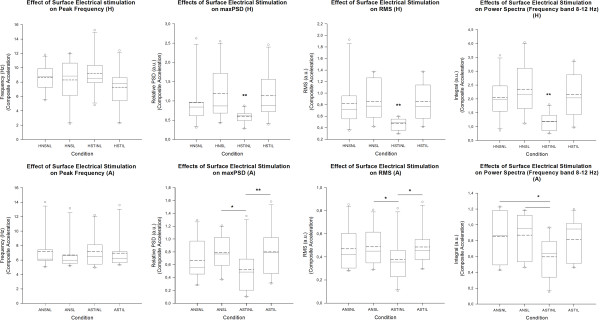
**Tremor parameters.** Box and whisker plots of peak frequencies (left panels), maximal power spectral density (maxPSD; middle left panels), RMS (root mean square; middle right panels), integral of the frequency band 8-12Hz (right panels) for composite data. Tremor recorded at the index finger (accelerometer 1) in the 4 conditions, during tasks H (Top panels) and A (Bottom panels). Continuous line: median; dashed line: mean values. Outliers: 5th and 95th percentiles. N = 10 subjects. Frequency peak: no significant changes. maxPSD: values in the condition HSTINL are significantly lower as compared to the 3 other conditions (**: p = 0.005); values in ASTINL are significantly lower than values in ANSL (*: p < 0.05) and in ASTIL (**: p < 0.01). RMS: in H, the lowest values occur in HSTINL (as compared to the 3 other conditions; **: inter-group difference p < 0.001); in A, values are significantly lower in ASTINL as compared to ANSL and ASTIL (*: p < 0.05). Integral 8-12Hz: in H, the lowest values are found in HSTINL (as compared to the 3 other conditions; **: inter-group difference p < 0.001); in A, values are significantly lower in ASTINL as compared to ANSNL and ANSL (*: p < 0.05). Frequencies are expressed in Hz; maxPSD, RMS and integrals of subbands are expressed in arbitrary units (a.u.).

The group analysis showed no significant change between the 4 conditions for the PFr of the most relevant accelerometer (accelerometer 1) (see left panels in Figure 3 - H: absence of stimulation effect p = 0.24; A: absence of stimulation effect p = 0.14). For the maxPSD, a significant stimulation effect was found for task H (see Figure 3, middle left panels). The multiple comparison procedure confirmed that maxPSD in condition HSTINL was significantly smaller (p = 0.005) as compared to the 3 other conditions. Statistical analysis of PSD of composite acceleration showed a clear decrease of the maxPSD to 62.18% during the condition HSTINL as compared to HNSNL (p = 0.01). Interestingly, the condition of augmented visual feedback blocked the beneficial effect of stimulation on tremor amplitude (HSTINL vs HSTIL: p = 0.01). We found a statistically significant interaction between augmented visual feedback and FES (interaction stimulation x vision: p = 0.039). Similar observations were made for the task A (Figure 3 Bottom). The analysis of the RMS of composite acceleration in task H confirmed the beneficial effect of electrical stimulation on tremor magnitude (stimulation effect: p < 0.001) and the blocking of this effect by augmented visual feedback (see Figure 3, middle right panels). Again, similar observations were made for the task A. No significant stimulation effect of FES on CF was found for task H (p = 0.16), but augmented visual feedback increased significantly the CF from 0.192 ± 0.04 (ANSNL) to 0.225 ± 0.05 (ANSL) (inter-group difference: p = 0.003, with a significant vision effect confirmed by post-hoc analysis with p < 0.05). The analysis of the effects of FES on frequency bands showed a significant effect on the 8-12 Hz band for task H (see Figure 3, right panels; highly significant inter-group difference with p < 0.001). Post-hoc multiple comparison procedure showed that the condition HSTINL was significantly different than the 3 other conditions. In task A, the condition of stimulation without augmented visual feedback reduced significantly the power spectra in the frequency band 8-12 Hz (inter-group comparison: p = 0.016; post-hoc analysis ANSNL vs ASTINL, and ANSL vs ASTINL: p < 0.05 ), and this effect was antagonized by vision (post-hoc analysis for the inter-group comparison between ASTINL and ASTIL: p < 0.05). For each axis, there was no stimulation effect, no vision effect, and no stimulation by vision interaction on frequency dispersion (p >0.10; see Additional file 1).

### Relationship Intensity of current delivered/Reduction of PSD

**Figure 4:**
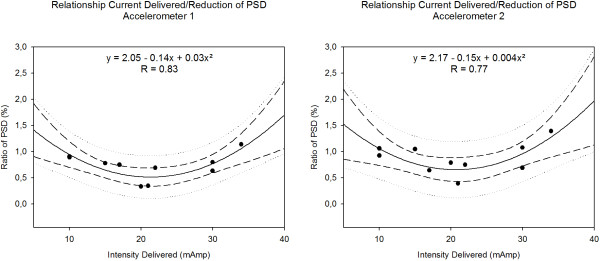
**Relationship of current delivered and FES effect.** The figure shows the relationship between the total amount of current delivered (axis x: sum of current delivered on the 4 muscles) and the effect of FES on reduction of PSD (expressed as% of baseline: ratio of the maxPSD in HSTINL by maxPSD in HNSNL; composite acceleration). Tremor recorded at the finger (accelerometer 1, left panel) and at the hand (accelerometer 2, right panel) The FES-induced decrease of PSD is stronger when the intensity of current delivered is around 20 mAmp. Dashed lines: 99% confidence interval; dotted lines: 99% prediction intervals.

We looked into the details of the relationship between the intensities of current applied on the skin and the reduction of tremor. We computed the sum of the intensity of current delivered at the 4 sites of skin stimulation. For accelerometer 1 (laser off), we found a bell-shaped profile in terms of reduction of tremor magnitude in the condition of stimulation -as compared to no stimulation- in task H (Figure 4). Fitting with a polynomial regression (2 factors: y = a + bx + cx^2^) was statistically significant at p = 0.017. A very similar observation was found for accelerometer 2, with a polynomial fit reaching a statistical significance of p = 0.04. FES-induced decreasing effect on maxPSD was the largest for the intensity of current delivered around 20 mAmp.

### Effect of FES on wrist stiffness

**Figure 5:**
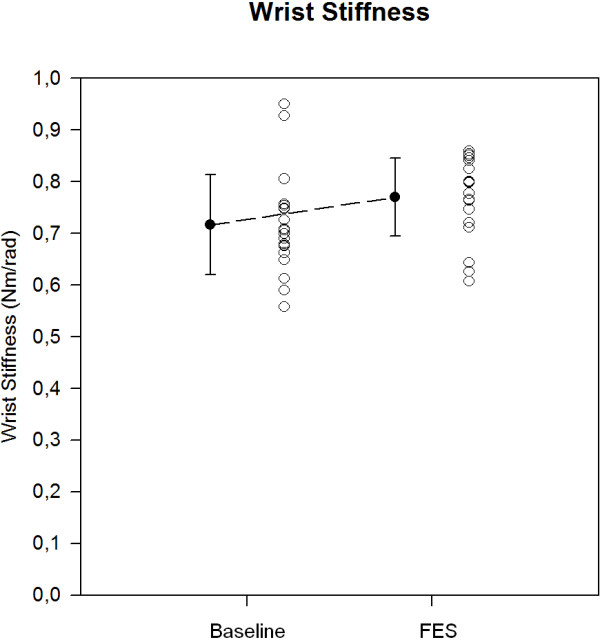
**Effects of FES on wrist stiffness in a single subject.** Stiffness is measured using a mechatronic haptic unit imposing slow extensions of the wrist (20 extensions at baseline and 20 extensions during FES). Individual values (open circles) and means (filled circles; +/- SD) are illustrated. Wrist stiffness is expressed in Nm/rad.

Mean values (+/- SD) of wrist stiffness before stimulation was 0,71 ±0,09 Nm/rad (Figure 5). During FES wrist stiffness was 0,77 ± 0,05 Nm/rad. The difference did not reach significance (p = 0.058; computation of the z score as compared to baseline gives a value of 0.56). The comparison of the last 10 trials of each condition (considered as the most representative given the adaptation of the subject to the manipulandum) showed a p value of p = 0.129, confirming an absence of effect on wrist stiffness.

### Effect of cerebellar Anodal cDCS on augmented visual feed-back effect

**Figure 6:**
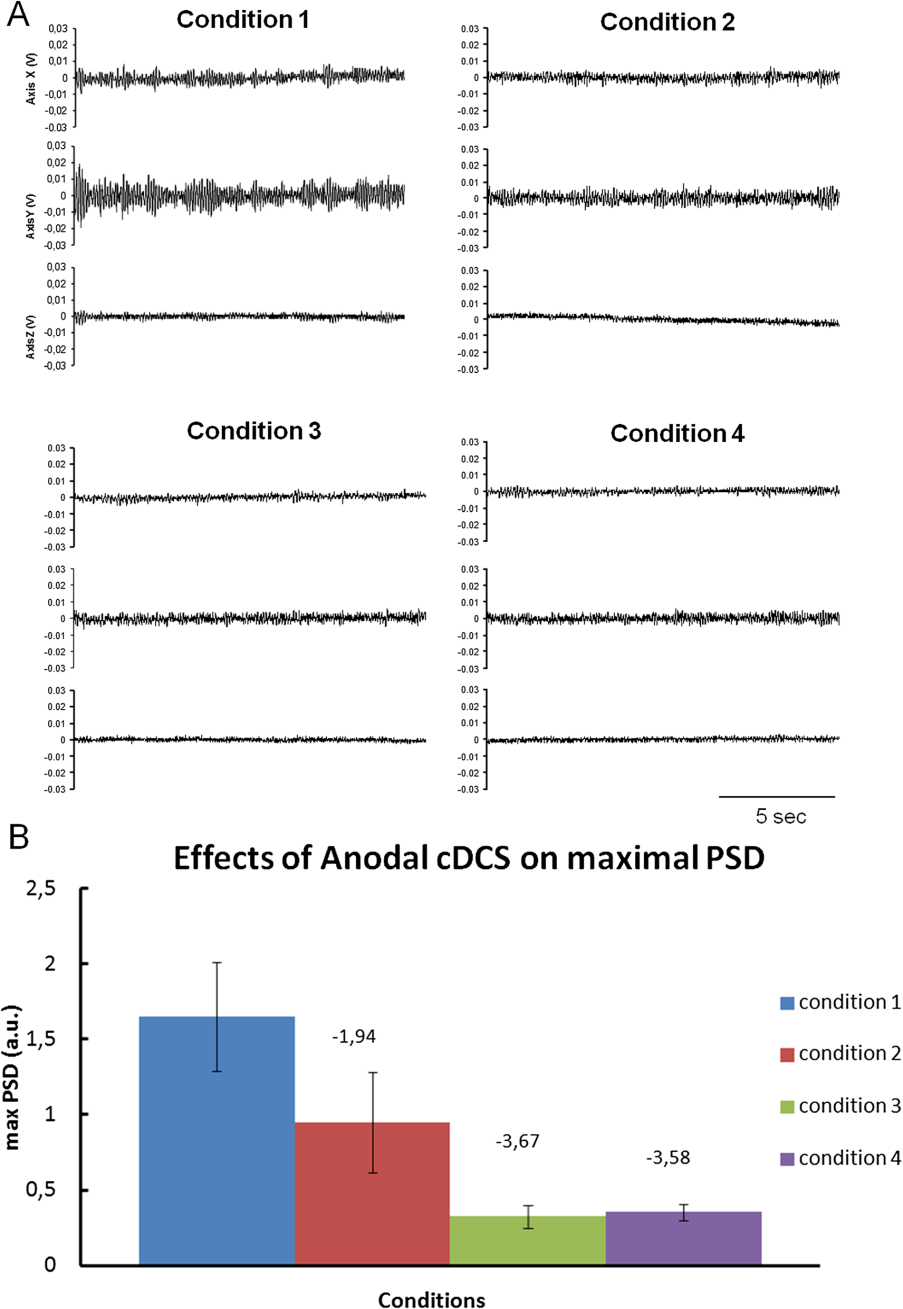
**Effect of Anodal cDCS of the cerebellum on physiological tremor in a single subject.** The 4 conditions refer to: (1): baseline, eyes open; (2) baseline, eyes closed, (3) post-Anodal cDCS eyes open, (4) post-Anodal cDCS eyes closed. Accelerometry traces in cartesian coordinates (from top to bottom: X, Y, Z axis, respectively; duration of epochs: 15 sec) for each condition are illustrated **(A)**. Mean values of maxPSD (+/- SD) are shown for the 4 conditions **(B)**. Values above the bars correspond to the number of SD between the mean of condition 1 and the mean of each column, respectively (z score as compared to baseline). Note the decrease of maxPSD with eyes closure at baseline and the subsequent reduction of maxPSD after Anodal cDCS of the cerebellum with disappearance of the vision effect.

Typical accelerometry traces are illustrated in Figure 6A. At baseline (before Anodal cDCS), maxPSD was greater in the condition eyes open (mean +/- SD: 1.65 +/- 0.36) as compared to the condition eyes closed (mean +/- SD: 0.95 +/- 0.33), as expected. The vision ratio (eyes closed/eyes open) was 57.5%. In the post-Anodal cDCS condition, the mean value of maxPSD dropped markedly to 0.33 +/- 0.08 in the condition eyes open. However, the augmented visual feed-back effect had disappeared: the mean value of maxPSD was 0.35 +/- 0.05. The vision ratio was now 108%. Similar observations were made for the RMS (see Additional file 1 for the other parameters studied).

## Discussion and conclusions

The novel findings of this study are the following: (1) FES induces a clear reduction of the magnitude of PT in upper limb, (2) the reduction is highly significant for tremor recorded at the index finger both when upper limb is maintained outstretched horizontally (H) and when motion of the hand is restricted to the wrist (A), (3) augmented visual feedback counteracts the beneficial effects of FES in terms of tremor reduction. We also show (indirectly: from the analysis of tremor frequency; directly: from the measurement of wrist stiffness) that FES does not impact on wrist stiffness and that the augmented vision effect is modulated by Anodal cDCS of the cerebellum.

Using a polynomial model at 2 factors, we found a relationship between the total amount of current delivered and the effect of FES on tremor reduction in our group of healthy subjects. The optimal intensity of current delivered occurs for middle range of intensities. The lowest intensities are associated with lower responses and the highest intensities decrease the performance of the FES technique probably by an overstimulation of the sensory nerve endings which cause an increase in the gain of reflex loops. Since the observation was made for both task H and task A, we can suggest that stimulation of the forearm muscles explains the effects observed. It would be very useful to compare these observations in selected categories of neurological patients. These relationships are likely impaired in neurological tremor associated with peripheral neuropathies, for instance, which induce a state of deafferentation and impair proprioception by affecting large sensory fibers. In these patients, vision is critically important to perform motor tasks, vision influencing motor representations [11].

Tremor signals recorded at index finger in task H strongly decreased in amplitude when FES was delivered in absence of contemporary augmented vision (HSTIL). The consistent reduction of maxPSD and Integral of 8-12 Hz band of frequency explains why we did not find any modification for the CF, which corresponds to the ratio maxPSD/Integral in a given frequency band. FES did not change the oscillations of PT in terms of peak frequency. Our results argue against an effect of FES on the mechanical properties (stiffness and inertia) of the musculo-skeletal system in healthy subjects since (1) such changes would impact directly on tremor frequency in a homogeneous sample of subjects and (2) a simultaneous increase in stiffness and inertia is extremely unlikely on the basis of our current knowledge of the biomechanical models of upper limb tremor [20]. The data obtained with the myohaptic technique also argue against an effect of FES on wrist stiffness. However, the situation might be different in neurological patients, since many neurological disorders impact on stiffness of joints and the upper limb’s responses to additional inertia are aberrant [6,23]. In addition, higher intensities of currents delivered on the skin might still modify stiffness, but unfortunately they would generate discomfort or pain. This would prevent their application during daily life.

What are the possible explanations for the beneficial effects of FES in PT? Pathways responsible for the generation of spinal cord segmental reflexes can be strongly influenced by feedback resulting from FES -as shown for instance by the long-lasting depression in the transmission of the soleus H reflex produced by FES applied to the rectus femoris muscle [31]. Other studies in healthy subjects suggest that FES increases the excitability of the cortex -or its connections to the spinal cord- effectively [32]. Investigations on the effects of FES of the common peroneal nerve during walking has revealed increased cortical excitability accompanied by an unchanged cortical inhibition, suggesting that FES increases excitability of the cortex by exerting specific effects on subpopulations of cortical neurons [33]. In neurological patients affected by upper limb paresis, the effectiveness of FES, as add on therapy to voluntary training of the paretic upper limb, has been explained by the changes in the corticospinal excitability, as indirectly revealed by Transcranial Magnetic Stimulation (TMS) studies [34]. Functional remodelling of the central nervous system from an “afferent effect” of electrical stimulation has been considered amongst the mechanisms to explain the improvement of upper limb function in patients with chronic hemiplegia [35]. One plausible explanation from our observations is that FES would impact on the central-neurogenic components of tremor as a result of an efferent effect. The concept of a central effect of FES is strengthened by studies on transcutaneous electrical nerve stimulation for pain in patients with carpal tunnel syndrome. Using functional Magnetic Resonance Imaging (fMRI), decreased activities in motor-related areas such as primary motor cortex, supplementary motor cortex, secondary somatosensory cortices, prefrontal and temporal cortex have been found [36]. The anatomical substratum of tremor networks includes motor-related areas and associative areas (prefrontal area, temporo-parieto-occipital cortex, posterior parietal area) [2]. Therefore, a modulation of the cortical activity in the direction of a decrease of excitability could be a logical explanation for our findings of FES-induced decreasing of PT [36]. This is strengthened by our findings of reduction of tremor amplitude using Anodal cDCS of the cerebellum. This is also the first demonstration that a modulation of PT is feasible by acting on a key-structure of the so-called Guillain-Mollaret triangle (cerebello-rubro-olivary pathway) which includes the cerebellum and represents a major regulator for tremorogenesis in human [29]. We suggest that further studies with fMRI should be conducted to define the mechanisms of action of FES on the brain networks underlying PT.

Our results show that augmented visual feedback counteracts the effects of FES in task H and task A, erasing the FES effect on tremor magnitude. To explain these findings, we suggest a mechanism of competition between vision effect and FES effect, where augmented vision competes against the effects of FES on tremor modulation. Previous findings have demonstrated an effect of enhanced visual information (provided by a laser emission) on PT [37]. The enhancement of tremor in the distal effector derives from subjects’ attempts to reduce tremor at the finger by exerting greater motoneuronal control over the hand [37]. Visual feedback of a moving limb changes the excitability of the corticospinal pathways [38]. Recent studies even highlight the importance of vision of one’s own hand and self-related processing in the changes of excitability of the motor cortex under visual guidance [39]. The modulation of motor cortex excitability dependent on objects observation (the “affordance effect”) is detectable in healthy human subjects using TMS. This effect is elicited by the observation of everyday-life graspable objects on motor cortex of resting observers. It has been shown that objects’ vision determines an increased cortical excitability at about 120 milliseconds after their presentation. This modulation is specific to the cortical representations of synergic muscles [40]. Attentional circuits contribute to the changes in motor cortex excitability [41]. Indeed, tasks of augmented vision feedback require a marked attentional demand by the subject. Mental imagery is also associated with a corticospinal facilitation, independently of hand posture [11]. The facilitation is greater when hand posture is compatible with the imagined movement. Besides these consequences on motor cortex excitability, vision also exerts a detectable effect on the excitability of intrinsic spinal cord circuits. A temporary modification of cervical spinal network excitability occurring after completion of an upper limb visuomotor force-tracking task has been demonstrated, with an increased excitability of segmental spinal cord reflexes [42].Therefore, there are at least two sites where our antagonizing effect of vision could take place: the motor cortex and the spinal cord. With respect to the central effect hypothesis, FES would induce a de-activation of the cerebral networks involved in PT genesis, whereas augmented vision would increase cortical activation. Our results on the effect of Anodal cDCS of the cerebellum on PT contribute to this debate and point towards a central origin of the augmented vision effect. Indeed, Anodal cDCS of the cerebellum erases the vision effect. Augmented vision feed-back effect is thus modulated by the cerebellum. Anodal cDCS of the cerebellum increases the inhibition exerted by the cerebellum on the motor cortex, resulting in a decreased excitability [16,17], which is a plausible explanation for the loss of augmented vision effect. This hypothesis needs to be confirmed in a group of subjects.

Although our results show effectiveness of FES in reducing PT and thus supports its possible role as an emerging therapy for neurological tremor, our findings highlight that tasks performed under augmented visual feedback may compromise this therapeutical approach. This is particularly relevant in subgroups of neurological patients who strongly rely on their visual feedback to control their movements, such as stroke survivors whose segmental and supra-segmental reflexes are hyper-excitable. Stiffness is dependent on vision, especially in tasks requiring accurate movements [43]. It can also be anticipated that patients exhibiting tremor in context of a cortical myoclonus enhanced by vision might also be a category not responsive to FES. Indeed, these patients are particularly sensitive to intense visual stimulation [44]. Clinical studies are required to address these issues.

## Abbreviations

A: Task A; ANOVA: Analysis of variance; cDCS: Continuous direct current stimulation; CF: Crest factor; ERC: Extensor radialis carpi; FFT: Fast fourier transform; FRC: Flexor radialis carpi; FES: Functional electrical stimulation; fMRI: Functional magnetic resonance imaging; H: Task H; MCT: Mechanical counter test; MCT-A: Mechanical counter test- alternate; MCT-U: Mechanical counter test-unilateral; NSNL: Stimulation off and laser off; NSL: Stimulation off and laser on; PT: Physiological tremor; PSD: Power spectral density; maxPSD: Maximal power spectral density; PFr: Peak frequency of power spectra; RMS: Root mean square; STIL: Stimulation on and laser on; STINL: Stimulation on and laser off; TMS: Transcranial magnetic stimulation.

## Competing interests

Mario Manto is supported by the Fonds National de la Recherche Scientifique Belgium.

## Authors’contributions

GG, MM: design of the study. FA, GG, MM: recordings. FA, GG, MM: data analysis. GG, MM: data interpretation. GG, MM: writing. All authors read and approved the final manuscript.

## Supplementary Material

Additional file 1 **Tremor parameters results: data related to accelerometers 2 (for tasks H and A) and 3 (for task H) in the 4 conditions (NSNL, SNL, STINL, STIL).** FES effect and vision effect on frequency dispesion for task H and A (accelerometers 1 and 2). Effect of Anodal cDCS of the cerebellum on physiological tremor: RMS data. Click here for file
