# Supplementary material for: Augmented visual feedback counteracts the effects of surface muscular functional electrical stimulation on physiological tremor
Source: J Neuroeng Rehabil. 2013 Sep 24;10:100. doi: 10.1186/1743-0003-10-100 (PMC3852972; doi:10.1186/1743-0003-10-100)
Supplement: Additional file 1 — Tremor parameters results: data related to accelerometers 2 (for tasks H and A) and 3 (for task H) in the 4 conditions (NSNL, SNL, STINL, STIL). FES effect and vision effect on frequency dispesion for task H and A (accelerometers 1 and 2). Effect of Anodal cDCS of the cerebellum on physiological tremor: RMS data. [file 1743-0003-10-100-S1.doc]

**Supplementary files**


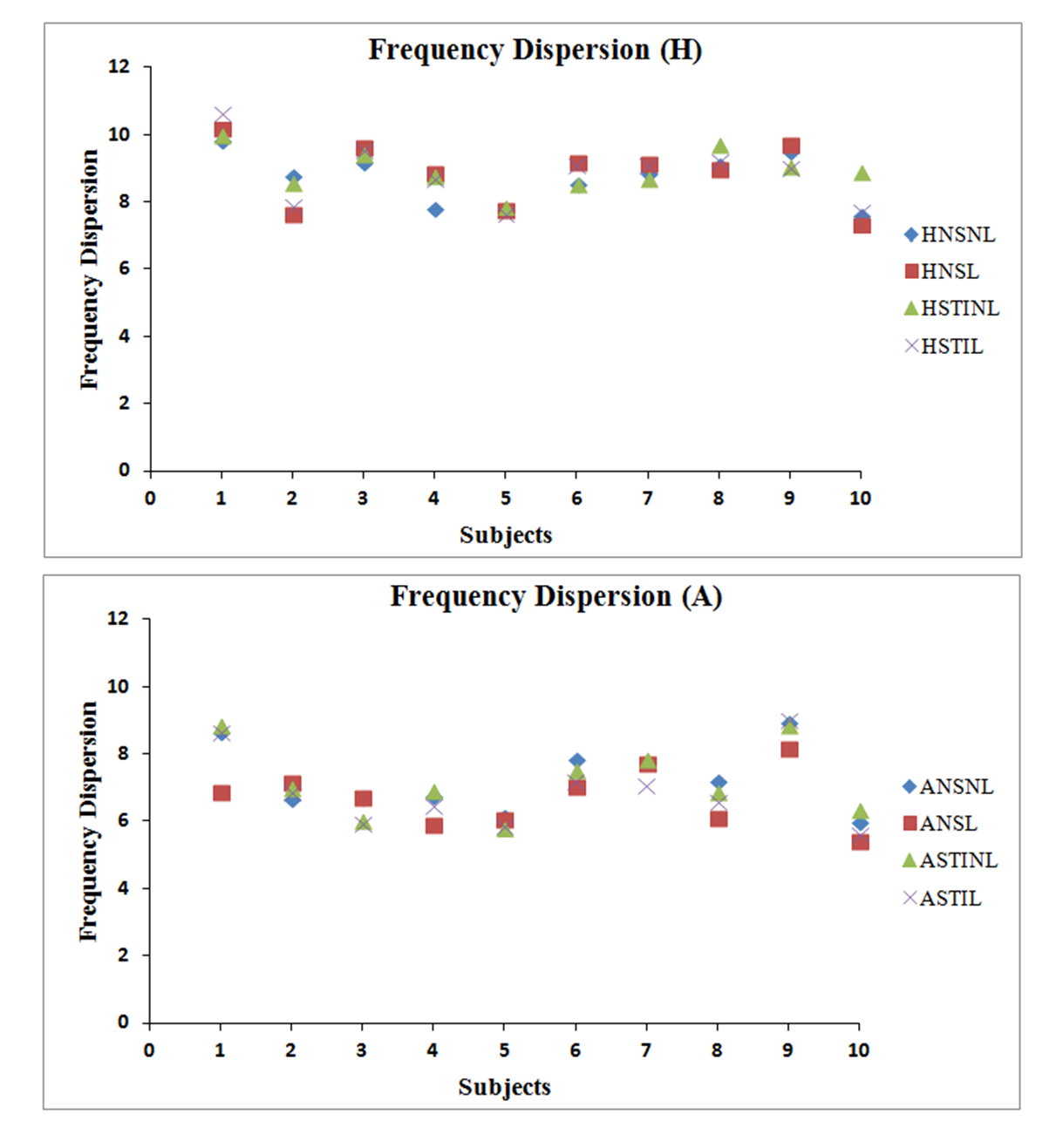


The graphs show the frequency dispersion in the 10 subjects in the 4 conditions (NSNL, SNL, STINL, STIL) for task H (Top) and task A (Bottom). Subjects present an overlap in the frequency dispersion’s values for the 4 experimental conditions.


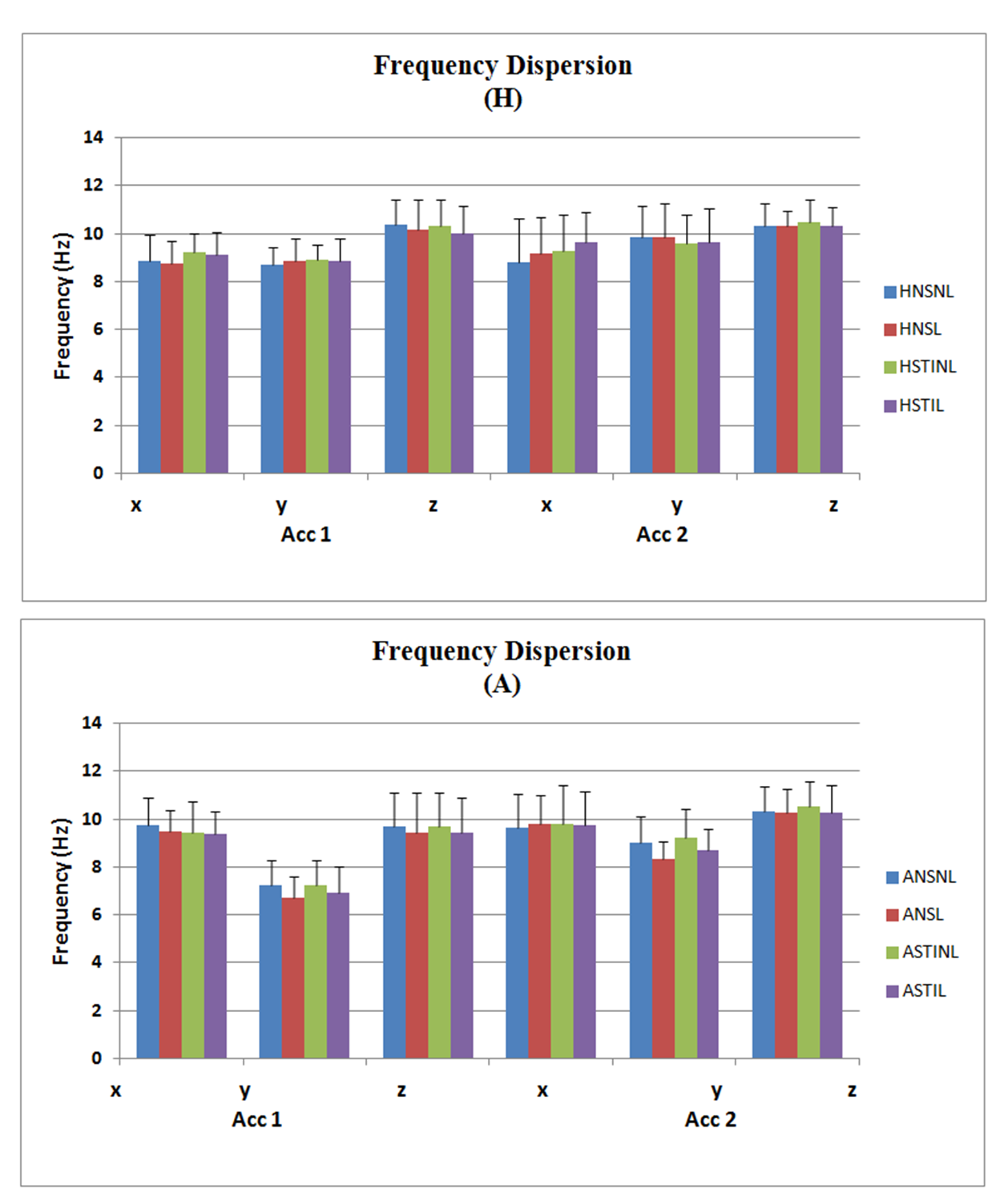


Frequency dispersion in healthy subjects (mean of 10 subjects; error bar: sd). Tremor recorded at the finger (acc 1) and at the hand (acc 2), using cartesian coordinates (x.y.z). No change in frequency dispersion in the 4 experimental conditions (NSNL.SNL.STINL.STIL). Top: task H. Bottom: task A.

| **Table 2. Composite Acceleration Data (H).** | | | | |
| --- | --- | --- | --- | --- |
| **Accelerometer** | **Condition** | **PSD** | **Integral 8-12 Hz** | **RMS** |
| **2** | **HNSNL** | 0.72±0.67(0.51) | 1.28±0.74(1.01) | 0.58±0.35(0.43) |
|  | **HNSL** | 0.79±0.75(0.53) | 1.43±0.89(1.23) | 0.57±0.27(0.48) |
|  | **HSTINL** | 0.58±0.52(0.41)  **^*^**vs HNSL (p=0.03) | 0.96±0.46(0.86)  **^*^**vs HNSL (p=0.002) | 0.44±0.2 (0.39)  **^*^**vs HNSL (p=0.004)  **^*^**vs HSTIL (p=0.004) |
|  | **HSTIL** | 0.77±0.63(0.60) | 1.30±0.63(1.18) | 0.58±0.27(0.52) |
| **3** | **HNSNL** | 0.60±0.29(0.53) | 1.27±0.55(1.09) | 0.55±0.36(0.45) |
|  | **HNSL** | 0.63±0.45(0.54) | 1.36±0.62(1.27) | 0.51±0.16(0.49) |
|  | **HSTINL** | 0.50±0.23(0.44)  **^*^**vs HSTIL (p=0.02) | 1.07±0.43(0.92)  **^*^**vs HNSL (p=0.004)  **^*^**vs HSTIL (p=0.004) | 0.45±0.13(0.44)  **^*^**vs HSTIL (p=0.006) |
|  | **HSTIL** | 0.62±0.32(0.53) | 1.31±0.55(1.29) | 0.54±0.19(0.53) |

Values are mean ± sd. Median values are given into brackets.

| **Table 3. Composite Acceleration Data (A).** | | | |
| --- | --- | --- | --- |
| **Condition** | **PSD** | **Integral 8-12 Hz** | **RMS** |
| **ANSNL** | 0.40±0.27(0.33) | 0.69±0.32(0.67) | 0.34±0.13(0.31) |
| **ANSL** | 0.44±0.24(0.37) | 0.74±0.30(0.67) | 0.35±0.11(0.31) |
| **ASTINL** | 0.41±0.31(0.30) | 0.60±0.26(0.51)  **^*^**vs ANSL(p=0.003)  **^*^**vs ASTIL (p=0.02) | 0.34±0.13(0.30) |
| **ASTIL** | 0.47±0.35(0.36) | 0.71±0.33(0.57) | 0.36±0.13(0.32) |

Values correspond to accelerometer 2. Median values are given into brackets.


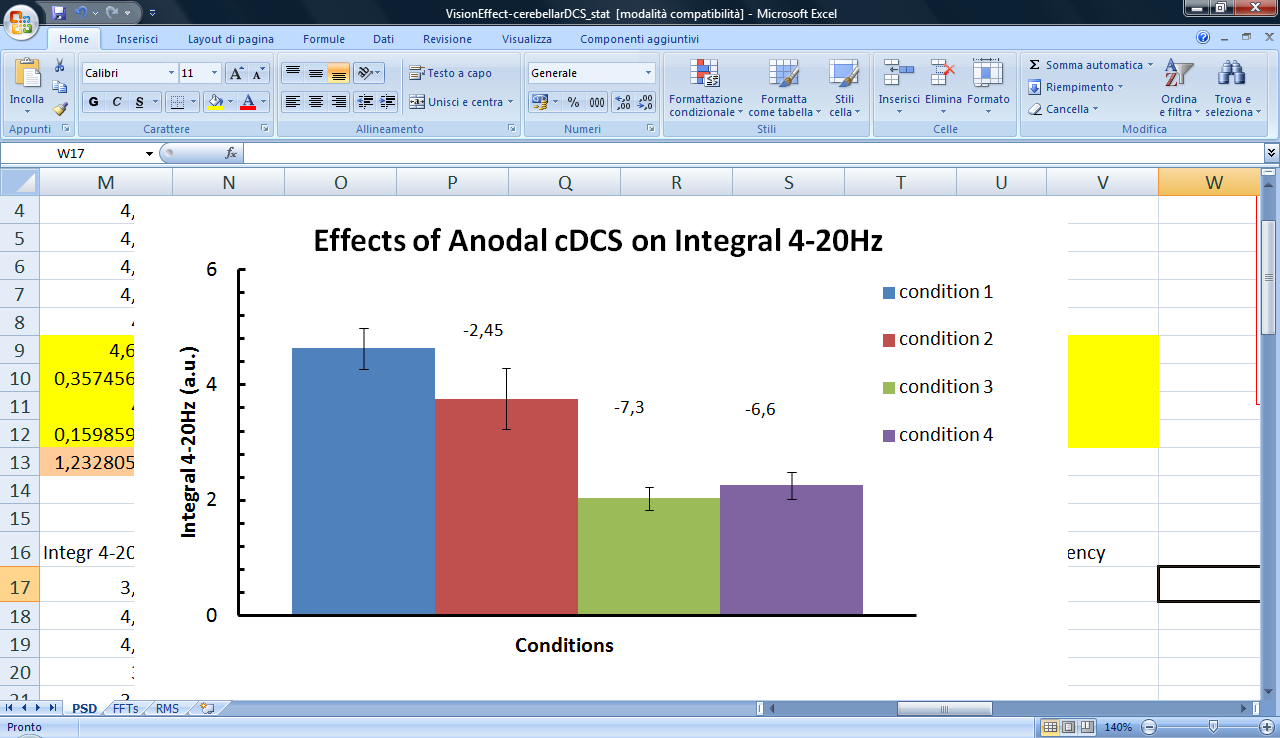


Effect of Anodal cDCS of the cerebellum on physiological tremor in a single subject. The 4 conditions refer to: (1): baseline, eyes open; (2) baseline, eyes closed, (3) post-Anodal cDCS eyes open, (4) post-Anodal cDCS eyes closed. Mean values of Integral in the 4-20 Hz frequency band (+/- SD) are shown for the 4 conditions: values above the bars correspond to the number of SD between the mean of condition 1 and the mean of each column, respectively (z score as compared to baseline). Note the decrease of Integral with eyes closure at baseline and the subsequent reduction after Anodal cDCS of the cerebellum with disappearance of the vision effect.
